# Circulating C-reactive protein levels as a prognostic biomarker in breast cancer across body mass index groups

**DOI:** 10.1038/s41598-024-64428-3

**Published:** 2024-06-24

**Authors:** J. B. Holm, E. Baggesen, D. Cronin-Fenton, J. Frystyk, J. M. Bruun, P. Christiansen, S. Borgquist

**Affiliations:** 1 Department of Oncology, Aarhus University Hospital, Aarhus, Denmark; 2 Department of Clinical Medicine, Aarhus University, Aarhus, Denmark; 3 Department of Clinical Epidemiology, Department of Clinical Medicine, Aarhus University Hospital, Aarhus, Denmark; 4 Department of Endocrinology, Odense University Hospital, Odense, Denmark; 5 Steno Diabetes Center Aarhus, Aarhus University Hospital, Aarhus, Denmark; 6 Department of Plastic and Breast Surgery, Aarhus University Hospital, Aarhus, Denmark

**Keywords:** Breast cancer, Obesity, Body mass index, Inflammation, C-reactive protein, Prognosis, Prognostic markers, Breast cancer, Cancer epidemiology, Breast cancer, Cancer epidemiology

## Abstract

Obesity and systemic inflammation are associated with breast cancer (BC) outcomes. Systemic inflammation is increased in obesity. We examined the association between C-reactive protein (CRP) and disease-free survival (DFS) and overall survival (OS) overall, and according to body mass index (BMI). We assembled a cohort of women with BC (stage I–III) seen at Aarhus University Hospital between 2010 and 2020 who donated blood at BC diagnosis (N = 2673). CRP levels were measured and divided into quartiles. We followed patients from surgery to recurrence, contralateral BC, other malignancy, death, emigration, or end-of-follow-up. We used Cox regression to estimate hazard ratios (HRs) with 95% confidence intervals (95% CIs) to compare outcomes across CRP quartiles, overall and stratified by BMI (normal-weight (18.5 ≤ BMI < 25 kg/m^2^), overweight (25 ≤ BMI < 30 kg/m^2^), and obesity (BMI ≥ 30 kg/m^2^)). During follow-up, 368 events (212 recurrences, 38 contralateral BCs, and 118 deaths) occurred (median follow-up 5.55 years). For DFS, high CRP (CRP ≥ 3.19 mg/L) was associated with an increased risk of events (HR_adj_:1.62 [95% CI = 1.14–2.28]). In BMI-stratified analyses, high CRP was associated with elevated risk of events in normal-weight and overweight (HR_adj_:1.70 [95% CI = 1.09–2.66]; HR_adj_:1.75 [95% CI = 1.08–2.86]), but in obesity, the estimate was less precise (HR_adj_:1.73 [95% CI = 0.78–3.83]). For OS, high CRP was associated with increased risk of death (HR_adj_:2.47 [95% CI = 1.62–3.76]). The association was strong in normal-weight and overweight (HR_adj_:3.66 [95% CI = 1.95–6.87]; HR_adj_:1.92 [95% CI = 1.06–3.46]), but less clear in obesity (HR_adj_:1.40 [95% CI = 0.64–3.09]). To sum up, high CRP levels at BC diagnosis were associated with inferior prognosis in early BC irrespective of BMI, although less clear in patients with obesity.

## Introduction

In 2020, 2.3 million women were diagnosed with breast cancer (BC) globally, making it the most prevalent cancer type (excluding non-melanoma skin cancer) in the world^1^. Despite 5-year survival rates approaching 90% in North America for patients with BC, nearly 700,000 women died from BC in 2020 worldwide^1,2^. Alongside, the prevalence of obesity (defined as a body mass index (BMI) ≥ 30 kg/m^2^) increased excessively worldwide, rising from 7 to 16% among women between 1975 and 2016^3^. Obesity is associated with an increased risk of developing at least 15 types of cancer, including postmenopausal BC^4^. Also, obesity is a prognostic disadvantage and is associated with shorter disease-free survival (DFS) and overall survival (OS) in BC^5,6^.

Inflammation is a hallmark of cancer^7^ and systemic inflammation is associated with poor BC prognosis^8,9^. Obesity is associated with low-grade inflammation^10^ and elevated levels of C-reactive protein (CRP)^11^, also among BC patients^12^. Thus, both obesity and systemic inflammation are associated with inferior BC outcomes. Yet, it is not clear if BC patients with obesity and high levels of systemic inflammation have a poorer prognosis compared with patients with obesity and lower levels of systemic inflammation.

CRP is an acute-phase protein released from the liver upon stimulation from cytokines such as interleukin 6^13^. CRP is part of the inflammatory cascade and a marker of systemic inflammation^14,15^. CRP levels are increased in people with obesity compared with their normal weight counterparts, and in cancer patients compared with healthy controls or patients with benign diseases^16^. A systematic review by Savioli et al. concluded that high pre-operative CRP levels were associated with an increased risk of BC-specific mortality and all-cause mortality^17^. Likewise, in a meta-analysis from 2011, Han et al. reported an association between elevated CRP levels and lower OS and DFS^18^. A meta-analysis by Mikkelsen et al. found that high CRP was an indicator of poor prognosis in metastatic BC, but the prognostic value in non-metastatic early BC could not be confirmed^19^.

As such, CRP levels may be prognostic in BC but only three studies have investigated this relationship across BMI groups, and they reported conflicting results^20–22^. Therefore, we investigated the prognostic potential of CRP in BC patients according to BMI groups. We hypothesized that higher circulating CRP levels were associated with poorer BC prognosis and that such an association was most pronounced in patients with obesity.

## Materials and methods

### Data sources

All data were merged through a unique identification number for each patient, linking all data with 100% accuracy. All patients with BC treated in Denmark are registered in the Danish Breast Cancer Group (DBCG) database^23^. The completeness of the DBCG database exceeds 95%^24^. From DBCG and through systematic investigation of medical records, we retrieved baseline data concerning patient-, tumor-, and treatment characteristics. All variables from DBCG were retrieved from “The Danish Clinical Quality Program—National Clinical Registries” (RKKP), which constitutes the infrastructure of the Danish clinical quality registries and the Danish Multidisciplinary Cancer Groups (DMCG)^25^. Information on emigration was retrieved through RKKP from the Civil Registration System^26^. Information on comorbidities was retrieved through RKKP from the National Patient Registry^27^. Information on height, weight, and thereof BMI was extracted from both medical records and the Danish Anesthesia Database (DAD)^28^. BMI was obtained by merging data from the medical records and the DAD. Regarding follow-up data, we extracted information based on a prespecified codebook. We reviewed all patient electronic medical records, which included pathological reports and digital imaging, to register all recurrences, contralateral BCs, other malignancies, and deaths.

### Study population

**Figure 1:**
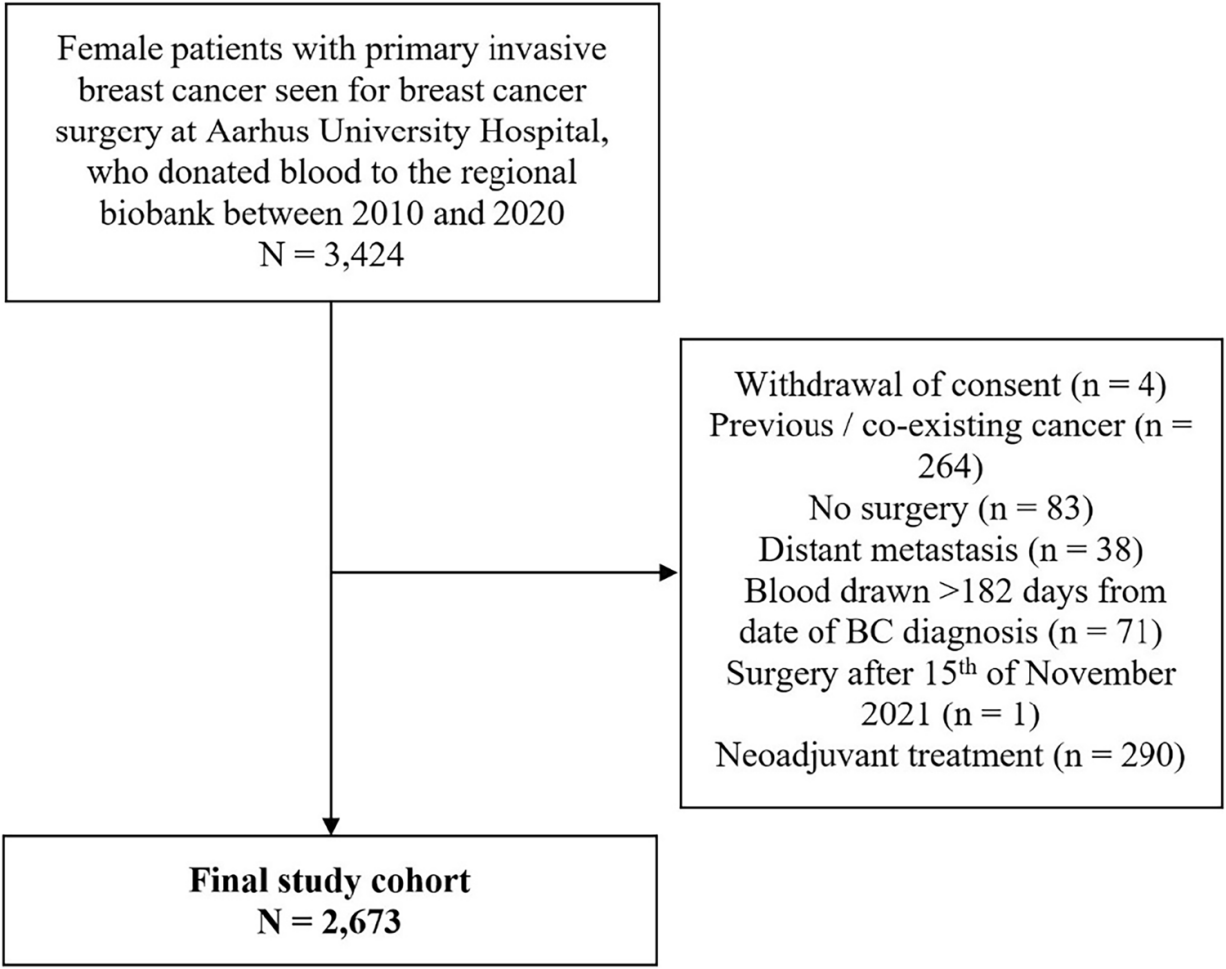
We identified 3424 female patients with primary invasive breast cancer who donated blood when seen for breast cancer surgery at Aarhus University Hospital between 2010 and 2020. We excluded patients who withdrew their consent. Patients with previous cancer or co-existing cancer (defined as a diagnosis of other malignancy before or within 90 days from the final primary surgery date) were excluded. Non-melanoma skin cancer was not classified as previous or co-existing cancer. As recurrence was an endpoint in the statistical analyses, patients who did not receive surgery or had distant metastasis at baseline were also excluded. Patients were excluded if a blood sample was drawn more than 6 months (182 days) before or after the date of invasive BC diagnosis. If the final primary surgery date was after the last date of follow-up (15th of November 2021) we excluded the patients. Finally, patients receiving neoadjuvant systemic therapy were excluded. After exclusion, 2673 patients were included in the statistical analyses. *BC* Breast cancer.

Our study cohort was women diagnosed with stage I-III BC between 2010 and 2020, who were referred for surgery at Aarhus University Hospital (AUH), for primary BC. At this department, all patients with BC were invited to donate blood for future research to the Regional Bio- and Genome Bank Denmark (RBGB)^29^ at the time of BC diagnosis. The blood was drawn between March 2010 and August 2020, median of seven days (interquartile range (IQR): 6–11 days) after the primary invasive BC diagnosis before breast surgery. Patients who received neoadjuvant systemic treatment were excluded from the analyses, as data on tumor characteristics are registered after neoadjuvant treatment and differ substantially from patients receiving up-front breast surgery. The final study population consisted of 2673 patients, as illustrated in Fig. 1.

### C-reactive protein analyses

After the blood draw, serum was separated and subsequently stored at − 80 °C at the RGBG. In late 2020, serum samples were identified and released for analyses at the Department of Clinical Biochemistry, AUH. The CRP levels were analyzed with a high-sensitivity CRP (hs-CRP) test (Siemens ADVIA® Chemistry XPT system with “ADVIA® Chemistry CardioPhase™ High Sensitivity C‑Reactive Protein (hsCRP)-reagents”)^30^. The lowest detectable level was 0.2 mg/L.

### Definitions of analytic variables

CRP levels were categorised in quartiles (CRP-Q1: $\le$ 0.60 mg/L, CRP-Q2: 0.61–1.33 mg/L, CRP-Q3: 1.34–3.18 mg/L, CRP-Q4: $\ge$ 3.19 mg/L). In the statistical analyses, the lowest CRP quartile (CRP-Q1) served as the reference. It should be noted that CRP below 3 mg/L is considered to be within the normal range^31^. BMI was grouped according to the World Health Organization (WHO) definitions^32^: underweight (BMI < 18.5 kg/m^2^), normal-weight (18.5 ≤ BMI < 25 kg/m^2^), overweight (25 ≤ BMI < 30 kg/m^2^), and obesity (BMI ≥ 30 kg/m^2^). The closest registered BMI measurement from the date of the blood sample was used. Registrations within 182 days before or after blood draw were included (median same date as blood draw, IQR: 0–2 days after date of blood draw).

Age and menopausal status (defined according to DBCG guidelines) refer to the time of primary invasive BC diagnosis. For tumor size and nodal status, we categorized patients in groups according to the American Joint Committee on Cancer Staging 8th edition^33^. The histological grade was classified using the standardization from the Nottingham Group^33^. The histological classification followed the WHO Classification of Breast Tumors 3rd Edition^34^. Tumors without registration of invasive ductal or lobular carcinoma were categorized as “Other.” Estrogen receptor (ER) status was classified as “Negative,” if tumor cells showed no ER expression, and “Positive” if 1% or more of the tumor cells expressed ER. Human Epidermal Growth Factor Receptor 2 (HER2) expression was defined as either “Positive” or “Negative” through a combination of immunohistochemistry and Fluorescence In Situ Hybridization (FISH)-ratio according to the American Society of Clinical Oncology guidelines^35^.

The type of breast surgery was categorized as “mastectomy” (including patients with mastectomy after lumpectomy) or “lumpectomy,” based on the final surgery for the primary BC. Adjuvant systemic treatment (endocrine therapy, HER2-targeted treatment, and chemotherapy) and radiotherapy were handled as intention-to-treat variables according to DBCG protocols.

Recurrence refers to any recurrent invasive BC in the breast, lymph nodes, or elsewhere in the body (apart from the contralateral breast) ≥ 3 months after the final primary surgery date (defined as the last date of surgery for the primary BC). The hierarchy for recurrence date was whatever came first in stating a recurrence in the pathological report, clinical charts, or imaging information. When defining the recurrence as local, regional, or distant, we followed the clinical guidelines from DMCG^36^. Registration of malignancy in the contralateral breast in the pathological report after final surgery was classified as contralateral BC. Other malignancies apart from non-melanoma skin cancer in the pathological report and death were registered, too.

### Follow-up and statistical analysis

*Disease-free survival (DFS).* Follow-up for DFS began on the date of final primary surgery and continued until the first of the following: BC recurrence, contralateral BC, death, other malignancy, emigration, or end-of-follow-up (15th November 2021). We treated BC recurrence, contralateral BC, and death as events. We censored patients at other malignancies, emigration, or end-of-follow-up. However, if recurrence, contralateral BC, or death occurred within 30 days after diagnosis of other malignancy, the event was included in the analyses.

*Overall survival (OS)*. Follow-up for all-cause mortality began at the date of final primary surgery and continued until the first of any death, emigration, or end-of-follow-up (15th November 2021). We treated any death as an event. We censored patients at emigration or end-of-follow-up.

We calculated person-years, number of events, and incidence rate per 1000 person-years (with a 95% confidence interval (95% CI)) for each CRP quartile. We used Cox regression models to estimate crude and adjusted hazard ratios (HRs) with 95% CIs for DFS and OS. Patients were followed for a maximum of 10 years in the regression models. We adjusted for patient-, tumor-, and treatment characteristics in the adjusted analysis. Only patients with complete data in all regressed variables were included (N = 2485). We included the following variables: age (continuous), menopausal status (dichotomous), comorbidities (Charlson Comorbidity Index (CCI)) (categorical), BMI (categorical), histological grade (categorical), histological classification (categorical), tumor size (categorical), nodal status (categorical), ER status (dichotomous), HER2 status (dichotomous), surgery type (dichotomous), intended adjuvant systemic treatment (dichotomous), and intended adjuvant radiotherapy (dichotomous).

To explore whether the association between CRP and outcomes differed across BMI groups, we performed DFS and OS analyses stratified for BMI groups as described above. In the stratified analyses, we created CRP quartiles within each BMI group. We adjusted for patient characteristics in the adjusted model. In the stratified analyses, patients with underweight were excluded. In the stratified analyses, we also presented Aalen-Johansen estimates on DFS (events: BC recurrence, contralateral BC, and death; competing risks: other malignancy; censoring points: emigration and end-of-follow-up) and Kaplan–Meier estimates on OS (events: death; censoring points: emigration and end-of-follow-up).

### Ethical approval

The study was conducted in accordance with relevant guidelines and legislation. All projects applying for samples at the RBGB need approval from the Danish Data Protection Agency and the Danish Council on Ethics. The conduction of the study is approved by the Danish Council on Ethics (no. 1-10-72-192-20) and registered as a scientific project at Region Midtjylland, Denmark (no. 1-16-02-299-20). Informed consent was obtained from all participants included in the study.

## Results

Our cohort included 2673 patients with a median age of 62 years at BC diagnosis (see Table 1). The median BMI was lowest in CRP-Q1 (BMI = 22.49 kg/m^2^) and highest in CRP-Q4 (BMI = 28.33 kg/m^2^). In CRP-Q4, more patients were postmenopausal, had higher CCI scores, and larger tumors compared to CRP-Q1. Chemotherapy was more often administered to patients in the CRP-Q1 compared with CRP-Q4. In total, 64 patients (2.39%) had underweight, 1265 (47.33%) had normal-weight, 818 (30.60%) had overweight, and 486 (18.18%) had obesity. The characteristics of the patients in the adjusted analyses (N = 2485) were similar to the characteristics of the 2673 patients in the crude analyses.

**Table 1:** Descriptive characteristics of women with breast cancer stage I-III referred for surgery at Aarhus University Hospital, Denmark for primary breast cancer. Patients were diagnosed with breast cancer and donated blood samples for future research between 2010 and 2020.

	Total	CRP-Q1	CRP-Q2	CRP-Q3	CRP-Q4
	N = 2673	$\le$ 0.60 mg/L	0.61–1.33 mg/L	1.34–3.18 mg/L	$\ge$ 3.19 mg/L
		N = 680	N = 657	N = 668	N = 668
Age, median (IQR)	62 (52–69)	58 (49–66)	63 (52–70)	64 (55–70)	64 (55–70)
Age (years), categories					
< 50	452 (16.91%)	187 (27.50%)	98 (14.92%)	80 (11.98%)	87 (13.02%)
50–59	661 (24.73%)	184 (27.06%)	162 (24.66%)	162 (24.25%)	153 (22.90%)
60–69	924 (34.57%)	196 (28.82%)	229 (34.86%)	253 (37.87%)	246 (36.83%)
$\ge$ 70	636 (23.79%)	113 (16.62%)	168 (25.57%)	173 (25.90%)	182 (27.25%)
Missing	0 (0%)	0 (0%)	0 (0%)	0 (0%)	0 (0%)
Body Mass Index (kg/m^2^), median (IQR)	24.95 (22.31–28.39)	22.49 (20.75–24.57)	24.49 (22.32–27.10)	25.81 (23.23–29.05)	28.33 (24.80–33.12)
Body Mass Index, categories (kg/m^2^)					
Underweight < 18.5	64 (2.39%)	31 (4.56%)	15 (2.28%)	7 (1.05%)	11 (1.65%)
Normal-weight $\le$ 18.5 to < 25	1265 (47.33%)	498 (73.24%)	338 (51.45%)	265 (39.67%)	164 (24.55%)
Overweight 25 ≤ to < 30	818 (30.60%)	123 (18.09%)	229 (34.86%)	251 (37.57%)	215 (32.19%)
Obesity ≥ 30	486 (18.18%)	20 (2.94%)	64 (9.74%)	137 (20.51%)	265 (39.67%)
Missing	40 (1.50%)	8 (1.18%)	11 (1.67%)	8 (1.20%)	13 (1.95%)
Menopausal status					
Premenopausal	598 (22.37%)	222 (32.65%)	133 (20.24%)	123 (18.41%)	120 (17.96%)
Postmenopausal	2046 (76.54%)	446 (65.59%)	516 (78.54%)	539 (80.69%)	545 (81.59%)
Missing	29 (1.08%)	12 (1.76%)	8 (1.22%)	6 (0.90%)	3 (0.45%)
Charlson Comorbidity Index					
0	346 (12.94%)	95 (13.97%)	89 (13.55%)	88 (13.17%)	74 (11.08%)
1–2 (mild)	1885 (70.52%)	519 (76.32%)	472 (71.84%)	457 (68.41%)	437 (65.42%)
$\ge$ 3 (moderate/severe)	442 (16.54%)	66 (9.71%)	96 (14.61%)	123 (18.41%)	157 (23.50%)
Missing	0 (0%)	0 (0%)	0 (0%)	0 (0%)	0 (0%)
Tumor size					
0–20 mm	1889 (70.67%)	508 (74.71%)	466 (70.93%)	460 (68.86%)	455 (68.11%)
21–50 mm	723 (27.05%)	159 (23.38%)	174 (26.48%)	194 (29.04%)	196 (29.34%)
> 50 mm	56 (2.10%)	12 (1.76%)	16 (2.44%)	12 (1.80%)	16 (2.40%)
Missing	5 (0.19%)	1 (0.15%)	1 (0.15%)	2 (0.30%)	1 (0.15%)
Lymph node metastases					
0	1656 (61.95%)	428 (62.94%)	404 (61.49%)	420 (62.87%)	404 (60.48%)
1–3	734 (27.46%)	180 (26.47%)	183 (27.85%)	187 (27.99%)	184 (27.54%)
4–9	180 (6.73%)	47 (6.91%)	42 (6.39%)	38 (5.69%)	53 (7.93%)
$\ge$ 10	82 (3.07%)	21 (3.09%)	20 (3.04%)	20 (2.99%)	21 (3.14%)
Missing	21 (0.79%)	4 (0.59%)	8 (1.22%)	3 (0.45%)	6 (0.90%)
Histological classification					
Ductal	2008 (75.12%)	510 (75.00%)	508 (77.32%)	486 (72.75%)	504 (75.45%)
Lobular	331 (12.38%)	92 (13.53%)	75 (11.42%)	92 (13.77%)	72 (10.78%)
Other	334 (12.50%)	78 (11.47%)	74 (11.26%)	90 (13.47%)	92 (13.77%)
Missing	0 (0%)	0 (0%)	0 (0%)	0 (0%)	0 (0%)
Histological grade					
N/A	164 (6.14%)	39 (5.74%)	38 (5.78%)	38 (5.69%)	49 (7.34%)
Grade 1	608 (22.75%)	164 (24.12%)	147 (22.37%)	137 (20.51%)	160 (23.95%)
Grade 2	1218 (45.57%)	291 (42.79%)	311 (47.34%)	308 (46.11%)	308 (46.11%)
Grade 3	635 (23.76%)	170 (25.00%)	151 (22.98%)	169 (25.30%)	145 (21.71%)
Missing	48 (1.80%)	16 (2.35%)	10 (1.52%)	16 (2.40%)	6 (0.90%)
ER status (% positive cells)					
0% (negative)	278 (10.40%)	71 (10.44%)	59 (8.98%)	82 (12.28%)	66 (9.88%)
1–100% (positive)	2380 (89.04%)	602 (88.53%)	596 (90.72%)	583 (87.28%)	599 (89.67%)
Missing	15 (0.56%)	7 (1.03%)	2 (0.30%)	3 (0.45%)	3 (0.45%)
HER2 status					
Negative	2336 (87.39%)	581 (85.44%)	580 (88.28%)	583 (87.28%)	592 (88.62%)
Positive	282 (10.55%)	82 (12.06%)	66 (10.05%)	71 (10.63%)	63 (9.43%)
Missing	55 (2.06%)	17 (2.50%)	11 (1.67%)	14 (2.10%)	13 (1.95%)
Final primary surgery^a^					
Mastectomy	881 (32.96%)	235 (34.56%)	216 (32.88%)	222 (33.23%)	208 (31.14%)
Lumpectomy	1778 (66.52%)	438 (64.41%)	438 (66.67%)	443 (66.32%)	459 (68.71%)
Missing	14 (0.52%)	7 (1.03%)	3 (0.46%)	3 (0.45%)	1 (0.15%)
Adjuvant radiotherapy^b^					
No	516 (19.30%)	127 (18.68%)	134 (20.40%)	122 (18.26%)	133 (19.91%)
Yes	2078 (77.74%)	529 (77.79%)	503 (76.56%)	531 (79.49%)	515 (77.10%)
Missing	79 (2.96%)	24 (3.53%)	20 (3.04%)	15 (2.25%)	20 (2.99%)
Endocrine therapy^b^					
No	512 (19.15%)	133 (19.56%)	118 (17.96%)	133 (19.91%)	128 (19.16%)
Yes	2082 (77.89%)	523 (76.91%)	519 (79.00%)	520 (77.84%)	520 (77.84%)
Missing	79 (2.96%)	24 (3.53%)	20 (3.04%)	15 (2.25%)	20 (2.99%)
Anti-HER2 treatment^b^					
No	2316 (86.64%)	578 (85.00%)	571 (86.91%)	582 (87.13%)	585 (87.57%)
Yes	282 (10.55%)	82 (12.06%)	66 (10.05%)	71 (10.63%)	63 (9.43%)
Missing	75 (2.81%)	20 (2.94%)	20 (3.04%)	15 (2.25%)	20 (2.99%)
Adjuvant chemotherapy^b^					
No	1287 (48.15%)	279 (41.03%)	328 (49.92%)	343 (51.35%)	337 (50.45%)
Yes	1307 (48.90%)	377 (55.44%)	309 (47.03%)	310 (46.41%)	311 (46.56%)
Missing	79 (2.96%)	24 (3.53%)	20 (3.04%)	15 (2.25%)	20 (2.99%)

*CRP* C-reactive protein; *Q1* Quartile 1; *IQR* Interquartile range; *N/A* Not applicable; *ER* Estrogen receptor; *HER2* Human Epidermal Growth Factor Receptor 2. a: Defined as the last breast surgery procedure for the primary breast cancer. b: All systemic treatment variables and radiotherapy are intention-to-treat variables based on protocol allocation according to the Danish Breast Cancer Group.

In DFS analyses, 368 clinical events occurred over 14,962 person-years (median follow-up time 5.55 years). In the mortality analyses, 298 deaths were recorded during 15,803 person-years (median follow-up time = 6.02 years).

Table 2 presents the estimated DFS hazard ratios across CRP quartiles. In total, 70 events occurred in 3980 person-years in CRP-Q1, 82 events in 3724 person-years in CRP-Q2, 100 events in 3744 person-years in CRP-Q3, and 116 events occurred during 3513 person-years in CRP-Q4. In the adjusted analyses, we found a positive association between CRP-Q4 and the risk of clinical events compared to CRP-Q1 (CRP-Q4, HR_adj_: 1.62 [95% CI = 1.14–2.28]). Supplementary Tables 1 and 2 present the estimated DFS hazard ratios across CRP quartiles in more adjusted models, and with other malignancy treated as event in Supplementary Table 2.

**Table 2:** C-reactive protein quartiles in relation to disease-free survival and overall survival in breast cancer patients.

	Person-years	Number of events	Incidence rate per 1000 person-years (95% CI)	Crude hazard ratio [95% CI] (N = 2673)	Adjusted hazard ratio [95% CI]^a^ (N = 2485)
Disease-free survival					
Q1	3980	70	17.59 (13.91–22.23)		
Q2	3724	82	22.02 (17.73–27.33)		
Q3	3744	100	26.71 (21.95–32.49)		
Q4	3513	116	33.02 (27.53–39.61)		
Total	14,962	368^b^			
Overall survival					
Q1	4157	39	9.38 (6.85–12.84)		
Q2	3932	66	16.79 (13.19–21.36)		
Q3	3949	88	22.28 (18.08–27.46)		
Q4	3764	105	27.90 (23.04–33.78)		
Total	15,803	298			

*95% CI* 95% Confidence interval; *Q1* Quartile 1; *BC* Breast cancer.

a: Adjusted for age, menopausal state, comorbidities, body mass index, histological grade, tumor size, lymph node metastases, HER2 status, estrogen receptor status, histological classification, surgery, systemic treatment, and radiotherapy. b: 212 recurrences, 38 contralateral breast cancers, and 118 deaths.

Table 2 shows the estimated mortality hazard ratios across CRP quartiles. In total, 39 deaths were recorded during 4157 person-years in CRP-Q1, 66 deaths in CRP-Q2 during 3932 person-years, 88 deaths during 3949 person-years in CRP-Q3, and 105 deaths in CRP-Q4 during 3764 person-years. In the adjusted analyses, CRP-Q4 was associated with higher mortality risk compared to CRP-Q1 (CRP-Q4, HR_adj_: 2.47 [95% CI = 1.62–3.76]). Supplementary Table 3 presents the estimated mortality hazard ratios across CRP quartiles in more adjusted models.

**Figure 2:**
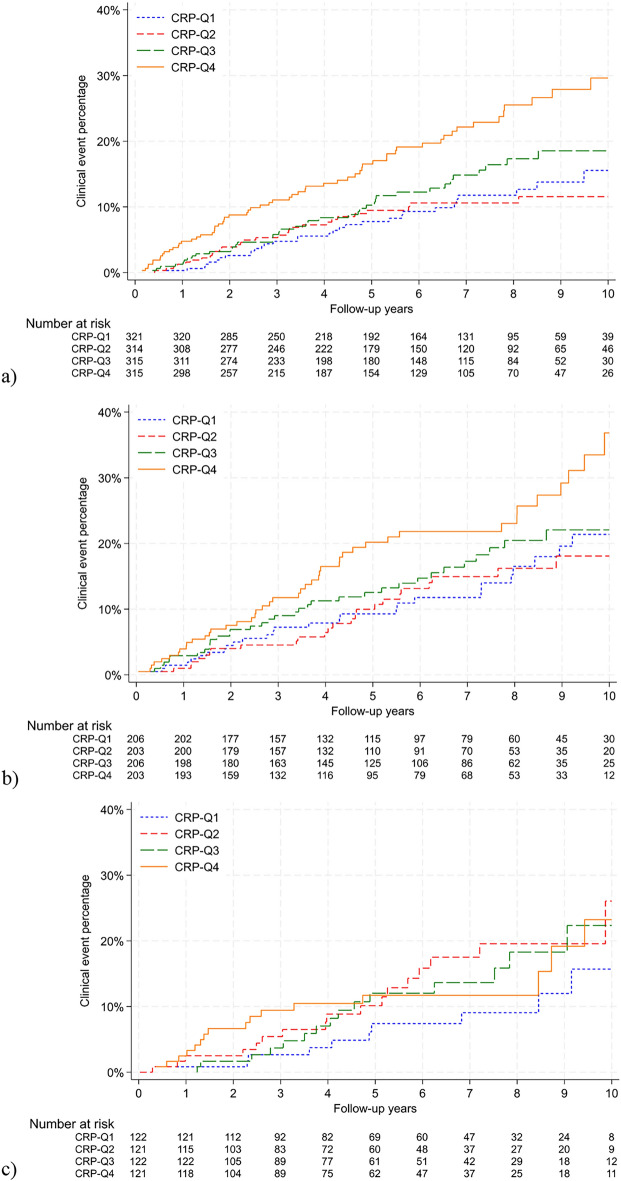
Cumulative incidences (Aalen–Johansen estimator) of clinical events (recurrence, contralateral breast cancer, and death) in women with breast cancer stage I–III according to C-reactive protein quartiles in patients with (**a**) normal-weight (body mass index 18.5- < 25 kg/m^2^) (CRP-Q1: ≤ 0.41 mg/L, CRP-Q2: 0.42–0.81 mg/L, CRP-Q3: 0.82–1.82 mg/L, CRP-Q4: ≥ 1.83 mg/L), (**b**) overweight (body mass index 25- < 30 kg/m^2^) (CRP-Q1: ≤ 0.85 mg/L, CRP-Q2: 0.86–1.62 mg/L, CRP-Q3: 1.63–3.26 mg/L, CRP-Q4: ≥ 3.28 mg/L), and (**c**) obesity (body mass index ≥ 30 kg/m^2^) (CRP-Q1: ≤ 1.75 mg/L, CRP-Q2: 1.77–3.55 mg/L, CRP-Q3: 3.56–7.02 mg/L, CRP-Q4: ≥ 7.07 mg/L). *CRP-Q1* C-reactive protein quartile 1 (lowest quartile); *CRP-Q4* C-reactive protein quartile 4 (highest quartile). The figures were made using Stata® version 18.

Figure 2 displays the cumulative incidences of clinical events (BC recurrence, contralateral BC, and death) across BMI groups. We saw an evident increase of incidences in CRP-Q4 compared to the other quartiles in BC patients with normal-weight or overweight, but not in patients with obesity.

Table 3 displays the estimated DFS hazard ratios according to CRP quartiles in BMI groups. We demonstrated an increased risk of an event among patients with CRP-Q4 as compared with CRP-Q1 in patients with normal-weight (CRP-Q4, HR_adj_: 1.70 [95% CI = 1.09–2.66]) and overweight (CRP-Q4, HR_adj_: 1.75 [95% CI = 1.08–2.86]). In patients with obesity, we found an increased risk of a clinical event in CRP-Q4 (CRP-Q4, HR_adj_: 1.73 [95% CI = 0.78–3.83]), though the precision of the estimate was less precise. It should be noted that the number of patients was lowest in the obesity group.

**Table 3:** C-reactive protein quartiles in relation to disease-free survival in breast cancer patients across body mass index groups.

	Person-years	Number of events	Incidence rate per 1000 person-years (95% CI)	Crude hazard ratio [95% CI]	Adjusted hazard ratio^a^ [95% CI]
Normal-weight				N = 1265	N = 1245
Q1 (N = 321) ($\le$ 0.41 mg/L)	1898	31	16.33 (11.49–23.23)		
Q2 (N = 314) (0.42–0.81 mg/L)	1846	29	15.71 (10.92–22.61)		
Q3 (N = 315) (0.82–1.82 mg/L)	1757	39	22.19 (16.21–30.37)		
Q4 (N = 315) ($\ge$ 1.83 mg/L)	1632	62	37.98 (29.61–48.72)		
Total (N = 1265)	7133	161			
Overweight				N = 818	N = 813
Q1 (N = 206) ($\le$ 0.85 mg/L)	1183	27	22.83 (15.65–33.29)		
Q2 (N = 203) (0.86–1.62 mg/L)	1142	25	21.88 (14.79–32.38)		
Q3 (N = 206) (1.63–3.26 mg/L)	1221	34	27.84 (19.89–38.96)		
Q4 (N = 203) ($\ge$ 3.28 mg/L)	1041	44	42.27 (31.46–56.80)		
Total (N = 818)	4587	130			
Obesity				N = 486	N = 482
Q1 (N = 122) ($\le$ 1.75 mg/L)	709	10	14.10 (7.59–26.21)		
Q2 (N = 121) (1.77–3.55 mg/L)	634	17	26.82 (16.67–43.15)		
Q3 (N = 122) (3.56–7.02 mg/L)	661	15	22.69 (13.68–37.63)		
Q4 (N = 121) ($\ge$ 7.07 mg/L)	643	16	24.89 (15.25–40.63)		
Total (N = 486)	2647	58			

*95% CI* 95% Confidence interval; *Q1* Quartile 1; *BC* Breast cancer.

a: Adjusted for age, menopausal state, and comorbidities.

**Figure 3:**
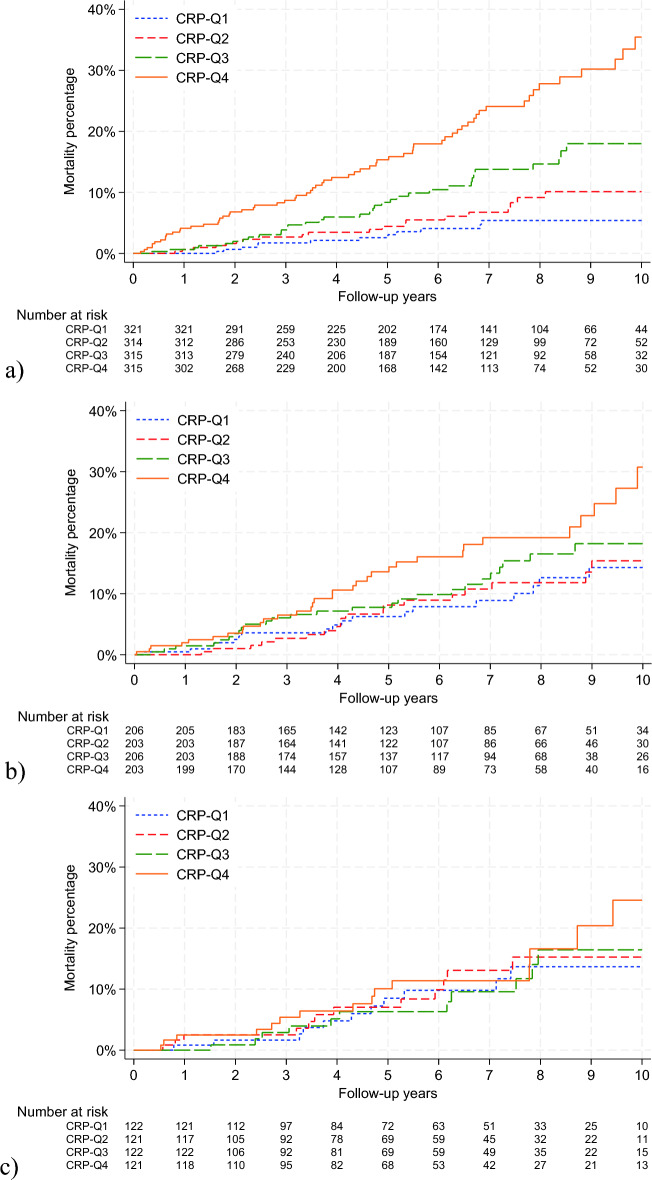
Cumulative incidences (Kaplan–Meier estimator) of deaths in women with breast cancer stage I–III according to C-reactive protein quartiles in patients with (**a**) normal-weight (body mass index 18.5- < 25 kg/m^2^) (CRP-Q1: ≤ 0.41 mg/L, CRP-Q2: 0.42–0.81 mg/L, CRP-Q3: 0.82–1.82 mg/L, CRP-Q4: ≥ 1.83 mg/L), (**b**) overweight (body mass index 25- < 30 kg/m^2^) (CRP-Q1: ≤ 0.85 mg/L, CRP-Q2: 0.86–1.62 mg/L, CRP-Q3: 1.63–3.26 mg/L, CRP-Q4: ≥ 3.28 mg/L), and (**c**) obesity (body mass index ≥ 30 kg/m^2^) (CRP-Q1: ≤ 1.75 mg/L, CRP-Q2: 1.77–3.55 mg/L, CRP-Q3: 3.56–7.02 mg/L, CRP-Q4: ≥ 7.07 mg/L). Abbreviations: *CRP-Q1* C-reactive protein quartile 1 (lowest quartile); *CRP-Q4* C-reactive protein quartile 4 (highest quartile). The figures were made using Stata® version 18.

Figure 3 shows the cumulative incidences of death across BMI groups. We observed a higher number of deaths in CRP-Q4 compared with the other quartiles among patients with normal-weight or overweight. In patients with obesity, we observed an increase in deaths in CRP-Q4 as well, becoming evident after eight years of follow-up.

Table 4 displays the estimated mortality hazard ratios according to CRP quartiles in BMI groups. In patients with normal-weight, being in the highest CRP quartile compared to the lowest CRP quartile was associated with an increased risk of death (CRP-Q4, HR_adj_: 3.66 [95% CI = 1.95–6.87]). In patients with overweight, an association was observed between CRP-Q4 and an increased risk of death compared to CRP-Q1 (CRP-Q4, HR_adj_: 1.92 [95% CI = 1.06–3.46]). In patients with obesity, we also observed an increased risk of death for patients in CRP-Q4 (CRP-Q4, HR_adj_: 1.40 [95% CI = 0.64–3.09]) compared with CRP-Q1, however, the precision of the estimate was weaker than in the other BMI groups.

**Table 4:** C-reactive protein quartiles in relation to overall survival in breast cancer patients across body mass index groups.

	Person-years	Number of deaths	Incidence rate per 1000 person-years (95% CI)	Crude hazard ratio [95% CI]	Adjusted hazard ratio [95% CI]^a^
Normal-weight				N = 1265	N = 1245
Q1 (N = 321) ($\le$ 0.41 mg/L)	1967	12	6.10 (3.46–10.74)		
Q2 (N = 314) (0.42–0.81 mg/L)	1917	20	10.43 (6.73–16.17)		
Q3 (N = 315) (0.82–1.82 mg/L)	1819	34	18.69 (13.35–26.16)		
Q4 (N = 315) ($\ge$ 1.83 mg/L)	1720	65	37.78 (29.63–48.18)		
Total (N = 1265)	7424	131			
Overweight				N = 818	N = 813
Q1 (N = 206) ($\le$ 0.85 mg/L)	1250	18	14.40 (9.08–22.86)		
Q2 (N = 203) (0.86–1.62 mg/L)	1243	19	15.29 (9.75–23.96)		
Q3 (N = 206) (1.63–3.26 mg/L)	1293	26	20.11 (13.69–29.54)		
Q4 (N = 203) ($\ge$ 3.28 mg/L)	1122	33	29.42 (20.92–41.39)		
Total (N = 818)	4907	96			
Obesity				N = 486	N = 482
Q1 (N = 122) ($\le$ 1.75 mg/L)	728	11	15.10 (8.36–27.27)		
Q2 (N = 121) (1.77–3.55 mg/L)	687	12	17.47 (9.92–30.76)		
Q3 (N = 122) (3.56–7.02 mg/L)	704	11	15.62 (8.65–28.21)		
Q4 (N = 121) ($\ge$ 7.07 mg/L)	685	15	21.89 (13.20–36.31)		
Total (N = 486)	2805	49			

*95% CI* 95% Confidence interval; *Q1* Quartile 1; *BC* Breast cancer.

a: Adjusted for age, menopausal state, and comorbidities.

## Discussion

This study demonstrated an association between high CRP levels and inferior outcomes in both DFS and OS analyses. In the BMI stratified analyses, we observed an association between high CRP and inferior DFS in patients with normal-weight, overweight, and obesity, although less evident among patients with obesity, which may be explained by low numbers of patients with obesity. In the OS analyses, we saw an over three-fold increased risk of death in patients with normal-weight and high CRP compared with low CRP. In patients with overweight, the increased risk of death was nearly two-fold, whereas a 40% increased risk of death was seen in patients with high CRP and obesity, but the precision of the estimate was lower than in normal-weight and overweight. We did not present BMI-stratified analyses for patients with underweight (BMI < 18.5 kg/m^2^), as the results were too imprecise due to the low number of patients with underweight.

Prior literature has shown inconsistent results regarding the prognostic value of CRP in BC. The meta-analysis by Mikkelsen et al. suggested a limited potential for CRP as a prognostic marker in non-metastatic settings^19^. In studies using CRP as a categorical variable, high CRP was associated with lower DFS and OS, but the estimates were imprecise. A Danish study of 2910 patients showed that the highest CRP tertile was associated with reduced OS and DFS^37^. The patients had BC stage I-IV diagnosed between 2002 and 2009, and blood was drawn at the time of diagnosis. In a cohort of BC patients with stage I-III disease in the United States (N = 2919), similar results were reported, in which blood was drawn at least 12 months after no evidence of disease (median 21.7 months)^38^. Higher all-cause mortality was observed in patients with CRP levels $\ge$ 10 mg/L compared to patients with CRP levels < 1 mg/L. A decrease in OS in pre-diagnostic CRP levels $\ge$ 10 mg/L compared to < 10 mg/L was found in a study by Wulaningsih et al.^39^ (N = 6606). Contrary to these studies, Frydenberg et al. found a decreased risk of all-cause death in the highest pre-diagnostic CRP tertile, and a similar correlation was found in DFS analyses (N = 192)^40^. Our findings are consistent with results from the larger studies^37–39^.

To our knowledge, only three studies have investigated the association between CRP and BC prognosis stratified by BMI ^20–22^. Our study is the first to explore the association with DFS across BMI groups. The latest study, a Chinese prospective multicenter cohort study with BC patients stage I-IV (N = 514) by Ruan et al.^20^, found a strong correlation between CRP > 10 mg/L and all-cause mortality. CRP > 10 mg/L was associated with lower OS in patients with BMI $\ge$ 24 kg/m^2^ and BMI < 24 kg/m^2^, however, the precision of the estimate was weaker in patients with BMI < 24 kg/m^2^ compared to patients with BMI $\ge$ 24 kg/m^2^. In 1114 BC patients (stage in situ to IV), Nelson et al. reported that only patients with higher CRP levels and normal-weight had an increased risk of death (HR: 1.39 [95% CI = 1.03–1.89]) for every 1 standard deviation increase in logCRP^21^. Patients with BMI > 25 kg/m^2^ and higher CRP levels had a slightly lower risk of death, but the precision of the estimate was low. In the NHANES III cohort, Wulaningsih et al. included 7780 females aged ≥ 20 without a cancer history at baseline^22^. A total of 44 BC deaths were reported. The risk of BC death per log CRP increase was higher in BMI < 30 kg/m^2^ compared to BMI $\ge$ 30 kg/m^2^ (HR 1.94 [95% CI = 0.51–7.29] vs. HR 1.40 [95% CI = 0.52–3.77]), however, the precision of the estimates was low.

Since our findings suggest that increased CRP across all BMI groups may be linked to worse BC prognosis, our results are similar to most of the results from the previous studies cited above. However, variations in study designs make a direct comparison of results difficult. Comparing our results with Ruan et al. is problematic since a BMI $\ge$ 24 kg/m^2^ includes both normal-weight, overweight, and obesity, and they used CRP as a binary exposure ^20^. Also, stage IV patients were included, the cohort was younger (mean 53.7 years), and treatment choice differed (i.e. only 5.3% received radiotherapy). It is not clear when Ruan et al. collected their blood samples. Our study cohort is larger, but based on a single-center cohort study. In the study by Nelson et al., the patients were older (mean age from 70.3 to 71.5 years in CRP quartiles), and they included patients with in situ and stage IV disease^21^. Blood samples were collected, on average 7.8 years before BC diagnosis which constitutes a major difference to our study^21^. Also, patients with underweight were included in the stratified analyses, and patients with CRP > 10 mg/L were excluded. Like Nelson et al., the CRP levels were measured before BC diagnosis by Wulaningsih et al.^22^. The authors had no information on BC incidence and disease characteristics^22^.

Our findings could have clinical implications. CRP at the time of diagnosis may be used by clinicians to identify BC patients with an increased risk of inferior outcomes. The precision of the estimate in patients with obesity could improve with a larger sample size, as our results in patients with obesity could be due to a type 2 error. However, many other factors are involved in the link between obesity and BC, such as adipokines and estrogens, as we previously reviewed^41^, and could potentially be of more significant importance than CRP for patients with obesity. Also, CRP is a surrogate marker for systemic inflammation and many factors (e.g. smoking and blood pressure) influence CRP levels^42^, and we were not able to take all these factors into consideration. Furthermore, CRP is not an appropriate marker for the local inflammatory environment in the breast.

Our study has limitations. First, it is a single-institutional study and the results may not apply to other institutions and countries. Second, we adjusted for potential confounders, but we cannot rule out the possibility of residual confounding, such as smoking status and alcohol consumption. Third, not all BC patients referred for surgery at AUH agreed to donate a blood sample, and we do not have information on the non-participants, which could lead to selection bias. Fourth, BMI is an indicator of general obesity but does not reveal information on body composition, which is important information as the inflammatory signatures differ between abdominal and gynoid obesity^43^. Fifth, we only measured CRP levels at a single time point, so we were unable to evaluate the impact of fluctuations in CRP, for example, due to acute infection or lifestyle factors.

## Conclusions

High circulating CRP levels at the time of BC diagnosis were associated with an inferior BC prognosis in this large Danish cohort. Furthermore, our study suggests that CRP may be a clinically relevant prognostic marker for BC prognosis across BMI groups. Future studies should investigate the relationship between CRP and BC in patients with obesity on a larger scale. Also, we encourage the investigation of other obesity-associated biomarkers in mapping the link between obesity and BC prognosis to identify patients in need of additional intervention.

### Supplementary Information

Supplementary Tables.

## Data Availability

The datasets generated and/or analyzed are not publicly available due to individual privacy could be compromised, but are available from the corresponding author on reasonable request.

## Abbreviations

AUH: Aarhus University Hospital
BC: Breast cancer
BMI: Body mass index
CCI: Charlson Comorbidity Index
CI: Confidence interval
CRP: C-reactive protein
DAD: Danish Anesthesia Database
DBCG: Danish Breast Cancer Group
DFS: Disease-free survival
DMCG: Danish Multidisciplinary Cancer Groups
ER: Estrogen receptor
FISH: Fluorescence In Situ Hybridization
HER2: Human Epidermal Growth Factor Receptor 2
HR: Hazard ratio
IQR: Interquartile range
OS: Overall survival
Q1: Quartile 1
RBGB: Regional Bio- and Genome Bank Denmark
RKKP: The Danish Clinical Quality Program—National Clinical Registries
WHO: World Health Organization
