# Supplementary material for: Circulating C-reactive protein levels as a prognostic biomarker in breast cancer across body mass index groups
Source: Sci Rep. 2024 Jun 24;14:14486. doi: 10.1038/s41598-024-64428-3 (PMC11196728; doi:10.1038/s41598-024-64428-3)
Supplement: Supplementary file 1 — Supplementary Tables. [file 41598_2024_64428_MOESM1_ESM.pdf]

Article title: Circulating C-reactive protein levels as a prognostic biomarker in breast cancer across body mass index groups

Journal name: Scientific Reports

Authors: Holm, J.B. (1,2), Baggesen, E. (2), Cronin-Fenton, D. (2,3), Frystyk J. (4), Bruun, J.M. (2,5), Christiansen, P. (2,6), Borgquist, S. (1,2)

Affiliations:

1: Department of Oncology, Aarhus University Hospital, Aarhus, Denmark

2: Department of Clinical Medicine, Aarhus University, Aarhus, Denmark

3: Department of Clinical Epidemiology, Department of Clinical Medicine, Aarhus University Hospital, Aarhus, Denmark

4: Department of Endocrinology, Odense University Hospital, Odense, Denmark

5: Steno Diabetes Center Aarhus, Aarhus University Hospital, Aarhus, Denmark

6: Department of Plastic and Breast Surgery, Aarhus University Hospital, Aarhus, Denmark

Corresponding author: Jonas Busk Holm, e-mail: jonasbuskholm@oncology.au.dk

Supplementary Table 1. C-reactive protein quartiles in relation to disease-free survival in breast cancer patients including three models in the adjusted analyses

	Person-years	Number of events	Incidence rate per 1000 person-years (95% CI)	Crude hazard ratio (95% CI) (N=2,673)	Model 1[a]: Adjusted hazard ratio (95% CI) (N=2,604)	Model 2[b]: Adjusted hazard ratio (95% CI) (N=2,508)	Model 3[c]: Adjusted hazard ratio (95% CI) (N=2,485)
Q1	3980	70	17.59 (13.91-22.23)	Reference	Reference	Reference	Reference
Q2	3724	82	22.02 (17.73-27.33)	1.25 (0.91-1.72)	1.10 (0.79-1.54)	1.00 (0.71-1.41)	1.01 (0.71-1.42)
Q3	3744	100	26.71 (21.95-32.49)	1.52 (1.12-2.06)	1.41 (1.02-1.95)	1.27 (0.91-1.77)	1.28 (0.91-1.79)
Q4	3513	116	33.02 (27.53-39.61)	1.88 (1.40-2.53)	1.73 (1.25-2.41)	1.68 (1.20-2.36)	1.62 (1.14-2.28)
Total	14962	368[d]					

Abbreviations: *95% CI* 95% Confidence interval; *Q1* Quartile 1.

a: Adjusted for age, menopausal state, comorbidities, and BMI. b: Adjusted for age, menopausal state, comorbidities, BMI, histological grade, tumor size, lymph node metastases, HER2 status, estrogen receptor status, and histological classification. c: Adjusted for age, menopausal state, comorbidities, BMI, histological grade, tumor size, lymph node metastases, HER2 status, estrogen receptor status, histological classification, surgery, systemic treatment, and radiotherapy. d: 212 recurrences, 38 contralateral BCs, and 118 deaths.

Supplementary Table 2. C-reactive protein quartiles in relation to disease-free survival in breast cancer patients including three models in the adjusted analyses treating other malignancy as event instead of censoring point

	Person-years	Number of events	Incidence rate per 1000 person-years (95% CI)	Crude hazard ratio (95% CI) (N=2,673)	Model 1[a]: Adjusted hazard ratio (95% CI) (N=2,604)	Model 2[b]: Adjusted hazard ratio (95% CI) (N=2,508)	Model 3[c]: Adjusted hazard ratio (95% CI) (N=2,485)
Q1	3980	94	23.62 (19.30-28.91)	Reference	Reference	Reference	Reference
Q2	3724	114	30.61 (25.48-36.78)	1.30 (0.99-1.70)	1.15 (0.87-1.53)	1.05 (0.79-1.41)	1.05 (0.78-1.41)
Q3	3744	133	35.52 (29.97-42.10)	1.51 (1.16-1.96)	1.37 (1.03-1.81)	1.26 (0.95-1.68)	1.26 (0.94-1.68)
Q4	3513	166	47.25 (40.59-55.02)	2.01 (1.56-2.59)	1.78 (1.34-2.35)	1.74 (1.30-2.32)	1.67 (1.25-2.24)
Total	14962	507[d]					

Abbreviations: *95% CI* 95% Confidence interval; *Q1* Quartile 1.

a: Adjusted for age, menopausal state, comorbidities, and BMI. b: Adjusted for age, menopausal state, comorbidities, BMI, histological grade, tumor size, lymph node metastases, HER2 status, estrogen receptor status, and histological classification. c: Adjusted for age, menopausal state, comorbidities, BMI, histological grade, tumor size, lymph node metastases, HER2 status, estrogen receptor status, histological classification, surgery, systemic treatment, and radiotherapy. d: 209 recurrences, 38 contralateral BCs, 146 other malignancies, and 114 deaths.

Supplementary Table 3. C-reactive protein quartiles in relation to overall survival in breast cancer patients including three models in the adjusted analyses

	Person-years	Number of deaths	Incidence rate per 1000 person-years (95% CI)	Crude hazard ratio (95% CI) (N=2,673)	Model 1[a]: Adjusted hazard ratio (95% CI) (N=2,604)	Model 2[b]: Adjusted hazard ratio (95% CI) (N=2,508)	Model 3[c]: Adjusted hazard ratio (95% CI) (N=2,485)
Q1	4157	39	9.38 (6.85-12.84)	Reference	Reference	Reference	Reference
Q2	3932	66	16.79 (13.19-21.36)	1.80 (1.21-2.67)	1.54 (1.02-2.33)	1.38 (0.90-2.11)	1.38 (0.90-2.12)
Q3	3949	88	22.28 (18.08-27.46)	2.39 (1.64-3.48)	2.19 (1.47-3.27)	1.97 (1.31-2.97)	1.99 (1.32-3.01)
Q4	3764	105	27.90 (23.04-33.78)	3.01 (2.08-4.34)	2.63 (1.75-3.94)	2.49 (1.65-3.78)	2.47 (1.62-3.76)
Total	15803	298					

Abbreviations: *95% CI* 95% Confidence interval; *Q1* Quartile 1.

a: Adjusted for age, menopausal state, comorbidities, and BMI. b: Adjusted for age, menopausal state, comorbidities, BMI, histological grade, tumor size, lymph node metastases, HER2 status, estrogen receptor status, and histological classification. c: Adjusted for age, menopausal state, comorbidities, BMI, histological grade, tumor size, lymph node metastases, HER2 status, estrogen receptor status, histological classification, surgery, systemic treatment, and radiotherapy.
